# The spontaneous emergence of rhythmic coordination in turn taking

**DOI:** 10.1038/s41598-022-18480-6

**Published:** 2023-02-24

**Authors:** Anna Zamm, Stefan Debener, Natalie Sebanz

**Affiliations:** 1 Department of Linguistics, Cognitive Science and Semiotics, Aarhus University, Aarhus, Denmark; 2 Department of Cognitive Science, Central European University, Vienna, Austria; 3 Institute for Psychology, University of Oldenburg, Oldenburg, Germany; 4 Cluster of Excellence Hearing4all, University of Oldenburg, Oldenburg, Germany

**Keywords:** Human behaviour, Social behaviour

## Abstract

Turn-taking is a feature of many social interactions such as group music-making, where partners must alternate turns with high precision and accuracy. In two studies of musical rhythm coordination, we investigated how joint action partners learn to coordinate the timing of turn-taking. Musically inexperienced individuals learned to tap at the rate of a pacing cue individually or jointly (in turn with a partner), where each tap produced the next tone in a melodic sequence. In Study 1, partners alternated turns every tap, whereas in Study 2 partners alternated turns every two taps. Findings revealed that partners did not achieve the same level of performance accuracy or precision of inter-tap intervals (ITIs) when producing tapping sequences jointly relative to individually, despite showing learning (increased ITI accuracy and precision across the experiment) in both tasks. Strikingly, partners imposed rhythmic patterns onto jointly produced sequences that captured the temporal structure of turns. Together, learning to produce novel temporal sequences in turn with a partner appears to be more challenging than learning to produce the same sequences alone. Critically, partners may impose rhythmic structures onto turn-taking sequences as a strategy for facilitating coordination.

## Introduction

Turn-taking is integral to many social interactions^1^, from conversational speech to group music-making. Turn-taking represents a unique coordination challenge: one partner’s actions cannot begin until the other’s ends. The ability to seamlessly transition turns is critical for joint actions such as choreographed group dance and music performance, where turns must follow a precise temporal—or rhythmic—structure. Experts in group music and dance can rhythmically alternate turns with seemingly little effort. However, many years of training are often required to achieve this feat. How joint action partners learn to coordinate the timing of turns in rhythmic action sequences like music is not well understood, as most scientific literature on action learning focuses on how individuals learn to perform actions. Several important questions remain to be answered: Do unique learning processes characterize how individuals learn to produce rhythmic action sequences in turn with a partner versus alone? And can turn-taking partners learn to achieve the same levels of temporal accuracy and precision as individuals producing the same rhythmic action sequence? In other words, can turn-taking partners learn to produce actions “as one”?

How individuals learn to produce rhythmic action sequences has been widely investigated, and the signatures of learning are well-defined. Individual learning is typically characterized by increases in the temporal accuracy and precision of action sequences relative to a target sequence^2–5^, where accuracy refers to the match between produced and target intervals between successive actions, and precision refers to the consistency of produced intervals. In addition to increases in precision and accuracy, individual learning is also often characterized by changes in error correction patterns over time^5,6^. Specifically, temporal errors at early learning stages are often serially dependent—meaning that they are correlated between successive actions—and then at later stages of learning, these serial dependencies are typically reduced^5^. These three signatures of learning—increased accuracy, precision, and reduced serial dependence of errors—have been observed across a range of repetitive actions, including paced finger-tapping (for a review see Repp and Su)^7^, reaching^8^, and postural sway^9^; these signatures have also been observed in patterns of spatial error in repetitive spatial tasks such as dart throwing^10^. Many theories of motor control have emerged to account for these learning signatures and to explain the error correction processes by which individuals optimize performance (for a review see Shadmehr, Smith, & Krakauer^33^ and Latash (2007)).

In contrast with individual motor learning, far less is known about the signatures or mechanisms of joint action learning. Only a handful of studies have investigated signatures of joint action learning: these studies reflect a diverse array of tasks (joint spatial coordination^11–16^, music duets^17,18^, rhythmic finger-tapping with a virtual partner^19^), research foci, and learning measures. Moreover, very few of these studies directly compare the signatures of individual and joint action learning for the same action sequence. Knoblich and Jordan^12^ made one of the few direct comparisons between individual and joint action action learning in a spatial coordination task. They found that partners’ learning rates were slower and accuracy was lower overall relative to individuals performing the same task alone, unless partners received auditory feedback about one another’s actions^12^. This finding was corroborated by van der Wel et at.^14,15^ who demonstrated that haptic feedback about a partner’s actions leads to the same learning outcomes for joint and individual learning of the same visuo-motor task. *Thus, sensory feedback from a partner’s actions, which provides information about a partner’s action timing, may be an important mechanism for facilitating acquisition of novel joint actions.*

In rhythmic joint actions such as music and speech conversation, the sensory outcomes of a partners’ actions often feature an inherent structure: Musical pitches unfold in an order defined by compositional rules of harmony, and speech prosody and syntax follow specific linguistic patterns. These structures are often highly predictable, which may facilitate temporal coordination above and beyond the mere presence of sensory feedback. For example, the ability to predict the pitch structure of a partner’s musical turns may facilitate one’s ability to predict when their turns will end and when to begin one’s own turns. Lappe et al.^20^ provide some evidence that learning to synchronize one’s actions with a metronome is facilitated by the presence of highly predictable sensory feedback^20^: However, it is unknown whether joint action learning is optimized when the sensory outcomes of a partners’ actions follow a predictable structure, or whether the mere presence of sensory feedback is sufficient for optimal learning.

The current study compared how individuals learn novel rhythmic auditory-motor sequences alone versus with a partner. The study focused specifically on how partners learn to take turns in rhythm, due to the high level of temporal coordination required of turn-taking partners and the paucity of literature on how partners learn to take turns. Three main questions were addressed: (1) Do the signatures of individual rhythmic sequence learning (increased temporal precision and accuracy, reduced serial dependency of timing errors) also characterize joint action learning? (2) Can partners achieve the same levels of temporal accuracy and stability as individuals over the same time-course of learning? (3) Does the predictability of sensory information associated with partners’ actions facilitate joint action learning?

## Methods

### Overview of studies

The above research questions were addressed in a pseudo-musical sequence learning paradigm for non-experts adapted from previous work^21^: Individuals with minimal musical training learned to tap at the rate of an initial metronome pacing cue either alone or in turn with a partner; each successive tap produced the next tone in a pitch sequence that was either predictable (a fixed melody) or random. Participants’ main task was therefore to learn to produce inter-tap intervals at the same rate as the metronome cue, and the pitch content served as a potential cue to facilitate coordination.

If the signatures of individual learning characterize joint action learning, then turn-taking partners should show increased temporal accuracy and precision, and reduced serial dependency of inter-tap-interval (ITI) errors across learning blocks; moreover, if the partners can learn to “act as one” then these signatures should reach the same levels in joint and individual learning conditions by the end of learning. If the predictability of sensory feedback about a partners’ actions facilitates joint action learning (see Pezzulo et al.^22^ for a theoretical account of use of sensory feedback from a partner during sensorimotor communication), then partners should show enhanced levels of coordination when learning to produce predictable relative to random pitch sequences. These hypotheses were tested in two joint action learning studies where partners took turns by alternating actions at higher (Study 1) or lower (Study 2) frequencies such that the temporal contingency between actions was either relatively high (Study 1) or low (Study 2).

### Study 1

#### Participants

48 participants (24 randomly-assigned dyads; 38 female; *M* age = 23.35 years, SD = 4.31 years) with less than six years of musical training (*M* musical training = 0.89 years, SD = 1.50 years, range = 0–5 years) were included in the current study sample. Less than 6 years of training was used as a criteria for inclusion, consistent with prior studies using a comparable threshold for defining musician populations (e.g., Scheurich et al.^21^; Livingstone et al.^23^; Zamm et al.^24^; Pfordresher et al. 2021), and also allowed for recruiting a sufficient sample size of individuals with minimal musical experience in Hungary, where students typically receive minimal musical education as part of their general studies. Participants were recruited through an online participant database (SONA systems, www.sonasystems.com) and through the local Marton Aron Iskolaszovetkezet Student Organization (https://www.mads.hu). All participants in the current sample reported basic English language skills ensuring the ability to understand experiment instructions, right-hand dominance, normal hearing, normal/corrected-normal vision, and no current use or history of psychiatric medication. Participants were screened for training on percussion instruments, which are known to enhance motor and perceptual timing abilities^25,26^. 4 additional dyads completed the study but were excluded from the current sample: 3 of these pairs were excluded due to technical issues during data acquisition; 1 pair was excluded due to a recruitment error. All participants provided informed consent prior to the experiment, and received gift vouchers or money for their participation. The study was approved by the United Ethical Review Committee for Research in Psychology (EPKEB) and was conducted in accordance with the Declaration of Helsinki (1991).

#### Stimuli

Two sets of 16-tone isochronous pitch sequences were created for two conditions in the current study: Predictable and Random. Predictable and Random sequences differed in two ways that maximized differences in pitch predictability: First, Predictable sequences were fixed across trials (same sequence on every trial), whereas Random sequences varied across trials (different sequence every trial). Second, Predictable sequences were originally composed to have a predictable harmonic structure, whereas Random sequences were generated by a random algorithm. Methods for generating these sequences are described in turn below. Both sets of sequences used tones drawn exclusively from the key of C-major pentatonic (C4,D4,E4,G4,A4, corresponding to MIDI pitches 60, 62, 64, 67, 69), a 5-tone scale featuring all pitches of the C-major scale except the 4th and 7th scale degrees (F,B), thereby eliminating dissonant intervals. The rationale for use of the C-major pentatonic scale was to ensure that random re-orderings of scale pitches introduced minimal changes in dissonance.

- *Predictable* 2 Predictable sequences were composed and counterbalanced across subjects. To generate these sequences, an initial sequence was composed to create an implied harmony that clearly evoked the key of C-major pentatonic, as verified by a trained music theorist: I-vii-I-IV-V-I-V-I- V-I-V-I-ii-I-V-I; pitch sequence = C4, D4, E4, A4, G4, E4, D4, C4, D4, E4, G4, E4, D4, E4, D4, C4). This sequence was then flipped from left to right (retrograde) to create a second sequence. The retrograde and initial sequences were used as the 2 Predictable sequences, and were equivalent with regards to pitch content.
- *Random* Random 16-tone pitch sequences were generated in MATLAB using the *randi* function, which generates column vectors of uniformly distributed random integers within a specified range. For each of the 16 sequence positions, *randi* generated 10,000 uniformly distributed random integers between 1 and 5, where integers between 1 and 5 represented the scale degrees of C-major pentatonic. The 16 resulting column vectors were horizontally concatenated to produce 10,000 16-integer sequences, where integers at each of the 16 sequence positions were drawn from a uniform random distribution of pitches in the C-major pentatonic scale. The resulting sequences were pruned for sequences containing the same pitch twice in a row and for sequences that did not feature all pitches in the pentatonic scale. This pruning procedure ensured that Predictable and Random pitch sequences featured the same number of unique pitches. 264 sequences remained after pruning. Each Random sequence was subsequently compared (note-by-note) with the two pre-composed Predictable sequences to confirm that the random sequence generation algorithm did not produce any sequences that were by chance identical to the pre-composed sequences.

#### Hardware and software

Two identical Akai Professional MAX25 keyboards (https://www.akaipro.com/max25) were used to record individual and dyadic finger tapping. Musical Instrument Digital Interface (MIDI) information (timing, pitch velocity) was sent from each keyboard over a separate channel; data from the two keyboards were merged via a MIDI merger (MIDI Solutions Inc., Canada), and sent via a MIDI-USB Interface (https://www.m-audio.com/products/view/uno to a Linux computer (Fedora 28, kernel 4.16.3–301.fc28.x86_64) running FTAP MIDI recording software^27^. The version of FTAP used was modified from the 2006 release; this modified version was validated in the first authors’ previous work^24^. FTAP sent a MIDI message via MIDI-USB to a tone generator (Roland SD50 Mobile Studio Canvas, Roland Corporation, Japan) for every keystroke received, which triggered the tone generator to produce the next pitch in a pre-programmed stimulus sequence. Each pitch was produced using the tone generator’s built-in piano timbre (GM2 Instrument Group 001, Piano-1, level = 100). FTAP was also used to generate metronome pacing sequences using the tone generator’s built-in woodblock timbre (Rhythm Group 001, Standard-1, level = 100). All piano tones and metronome clicks were programmed in FTAP to have a fixed MIDI velocity (127) and fixed duration. Audio from the tone generator was delivered to participants via headphones (Audio-Technica ATH-M50x Professional Monitor Headphones, Audio-Technica Corporation, United Kingdom) at maximum volume level (volume dial = 10). Each member of a dyad wore separate headphones; headphone pairs were connected to the tone generator phones input jack via a standard headphone splitter.

#### Experimental design

The current study implemented a 2 (Pitch Predictability, between-subject) × 2 (Task, within-subject) × 2 (Learning Block, within-subject) mixed factorial design.

**Figure 1:**
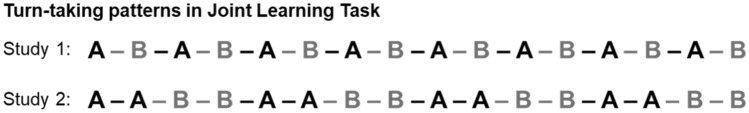
Turn-taking patterns in Joint task. The Joint task was implemented across 2 studies (Study 1 and Study 2), which differed primarily with regards to turn-taking structure, as shown above. Top panel: Turn-taking pattern in the Study 1 Joint task, Bottom panel: Turn-taking pattern in the Study 2 Joint task. In both panels, A (black) denotes tones produced by Partner A and B (gray) denotes tones produced by Partner B. Black dashes indicate inter-tone intervals resolved by Partner A and gray dashes indicate inter-tone intervals resolved by Partner B. In both studies, the partner who started each trial was counterbalanced across Learning Blocks to minimize the emergence of possible leader/follower dynamics.
- **Learning block** featured four levels corresponding to *blocks over which learning was expected to occur* (4 total Learning Blocks). Within each Learning Block, participants completed both tasks in a fixed order (counterbalanced across pairs), either Individual followed by Joint or Joint followed by Individual.

- **Pitch Predictability** was manipulated across 2 between-subject conditions: *Predictable* and *Random*. Participants in the Predictable condition, heard and produced stimulus pitch sequences that were predictable in two ways: (1) sequences featured a clear pre-defined pentatonic scale structure (see Stimulus section), (2) the same pitch sequence was produced on every trial of the experiment. The two pre-composed Predictable pitch sequences were counterbalanced across participants; partners within a pair received the same pitch sequence.
  Participants in the Random condition heard and produced pitch sequences that were random in two ways: First, sequences were generated with a random algorithm (see Stimulus section); second, a different random pitch sequence was produced on every trial of the experiment, ensuring that participants could not predict the pitch sequence across trials. Critically, both Predictable and Random conditions were drawn from the pentatonic scale and therefore did not differ with regards to frequency of consonant/dissonant intervals.
  Pitch predictability was implemented as a between-subjects variable for two reasons: (1) to minimize potential perception of structure in random sequences after hearing predictable sequences (e.g., 28) and (2) so that training effects from producing one condition did not carry over to the other condition.
- **Task (2 levels)** was manipulated across 2 conditions: *Individual* and *Joint*. In the Individual condition, participants learned to produce stimulus pitch sequences alone by tapping on a single key on a piano keyboard; every keypress produced audio associated with the next tone in the melody. In the Joint condition, participants learned to produce stimulus pitch sequences with a partner; partners tapped in alternation (first partner 1, then partner 2, then partner 1, etc.; see Fig. 1) and each tap produced the next tone in the pitch stimulus sequence. Importantly, auditory outcomes were identical across Individual and Joint conditions, the only difference was whether the pitch sequence was individually or jointly produced.

### Procedure

Participants completed two musical sequence tasks upon arrival at the lab: An Individual task and a Joint task (order counterbalanced). In the Individual task, participants learned to produce pitch sequences alone, and in the Joint task, participants learned to produce pitch sequences with a partner (see above). These tasks were interleaved across 4 Learning Blocks. For both tasks the procedure was the same within each block, featuring a Listen phase, a Practice phase, and a Test phase:

- *Listen phase (< = 2 trials)* In the Listen phase of each task, participants heard a MIDI recording of either the same melody that they would subsequently produce (Predictable), or a pitch sequence that was generated using the same algorithm (Random). Pitch sequences were presented at a rate of 120 BPM (500 ms inter-onset interval), corresponding to the center of the range in which music is typically produced^28^.
  The experimenter instructed participants as follows (curly brackets enclose distinct instructions for Individual/Joint tasks, square brackets enclose distinct instructions for the Predictable/Random tasks):
  We will let you listen to [the melody that you will produce (Predictable) / a sample melody (Random)]. [The sample melody is in the same style and will sound like the melodies that you will produce (Random only)]. Please listen carefully, and use this as an opportunity to get an idea of how you should aim to sound when you {produce the melody yourself (Individual) / when you produce the melody together (Joint)}.
  Participants were allowed to hear up to two trials of the pitch sequence (1 trial = 1 sequence iteration). The goal of this Listen phase was to expose participants to an “ideal” performance of the pitch sequence with regards to timing and fluency.
- *Practice Phase (2 trials).* After the Listen phase, participants completed the Practice phase in which they practiced producing the same stimulus sequence they heard during Listen. Participants either produced the melody alone (Individual) or with a partner (Joint), depending on the task.
  Participants in the Individual Task were instructed as follows:
  Now you have the chance to produce this sample melody… you will produce this melody by tapping on the piano key; every time you tap you will hear the next tone in the melody. Your goal will be to produce the melody so that it sounds like what you heard during Listening. The tempo at which you should produce the melody will be indicated by a metronome cue at the beginning of each trial.
  Participants in the Joint Task were instructed as follows:
  Now you have the chance to produce this sample melody together…On each trial, Partner A will produce the first melody tone by tapping on their designated key, then Partner B will tap the next melody tone by pressing on their designated key, then Partner A will produce the third melody tone by tapping on their key, and so forth.
  Your goal will be to co-produce the melody so that what you co-produce sounds like what you heard during listening. The tempo at which you should take turns producing melody tones will be indicated by a metronome cue at the beginning of each trial.
  On each practice trial, participants heard 8 metronome clicks at a rate of 120 BPM (500 ms inter-onset interval, IOI), and were instructed to start tapping when the metronome stopped and to continue tapping until they heard no more sound. The sound on each trial cut out after participants produced 16 taps (see above). Participants were allowed up to two practice trials (1 trial = 1 iteration of the stimulus sequence); in the Joint task, practice trials were only counted if they reflected the correct (instructed) turn-taking pattern, and the experimenter gave feedback on accuracy after each trial.
- *Test Phase (9 test trials).* After the Practice phase, participants completed the Test phase. In this Test phase, participants produced either the same stimulus melody as during Practice (Predictable), or produced a melody generated using the same algorithm (Random). Instructions were identical to the Practice phase, except that participants in the Random condition were told that their taps would generate a different pitch sequence on each trial. Participants completed a total of 9 test trials; in the Joint task, only “correct” trials were counted towards this total, and participants had to continue the task until they produced 9 correct trials. The experimenter pointed out errors in turn-taking accuracy and did not comment on trials where the turn-taking was correct.

### Study 2

Turn-taking structure may influence how partners learn to coordinate joint actions. For example, partners may correct interval timing in a turn-taking sequence differently depending on whether the interval was jointly produced (occurred at a turn-transition) or individually produced (occurred within a turn). Study 1 could not test this possibility because all intervals were jointly produced. Study 2 replicated Study 1 but with a different turn-taking structure (see Fig. 1): Partners produce pitch sequences at a cued rate by alternating every two taps instead of every tap. Therefore, each Joint action sequence featured intervals that were both individually produced (occurred within a turn) or jointly produced (occurred between turns). The logic behind adopting this turn-taking structure was to allow for dissociating individual from joint error correction processes, which occur at different lags in the Study 2 structure. Study 2 used exclusively Random pitch sequences (same as Random Pitch condition from Study 1), as Study 1 did not yield an effect of Pitch Predictability (see Results).

#### Participants

24 participants (12 randomly-assigned dyads; 15 female; *M* age = 23.88 years, *SD* = 3.35 years; 23 right-handed, 1 ambidextrous) with less than 6 years of musical training (*M* musical training = 1.21 years, SD = 1.75 years, range = 0–5 years) were included in Study 2. The number of participants was determined based on the number of participants in each experimental group in Study 1. Participants were recruited through an online participant database (SONA systems, www.sonasystems.com) and through the local Marton Aron Iskolaszovetkezet Student Organization (https://www.mads.hu). All participants in Study 2 were recruited using the same criteria as Study 1. One additional pair was recruited but excluded from the study due to a technical issue during recording. All participants provided informed consent prior to the experiment, and received gift vouchers or money for their participation. The study was approved by the United Ethical Review Committee for Research in Psychology (EPKEB) and was conducted in accordance with the Declaration of Helsinki (1991).

#### Stimuli

Study 2 stimuli comprised the same pitch sequences as the Random condition in Study 1 (see above).

#### Design and procedure

Study 2 followed an identical Design and Procedure to Study 1, except that only Random pitch sequences were used (i.e., factors were Task x Learning Block, no Pitch Predictability factor), and partners observed a different turn-taking sequence during the Joint task (see Fig. 1). Specifically, partners in Study 2 alternated producing every two tones, such that the turn-taking sequence for the 16-tone was: A–A–B–B–A–A–B–B–A–A–B–B–A–A–B–B, where A = tone produced by partner A and B = tone produced by partner B. The partner who started each Joint learning trial was counterbalanced across learning Blocks. The rationale behind this change in turn-taking pattern was to determine whether the temporal patterning observed in Study 1 extends to different forms of turn-taking.

## Data processing and analysis

Data associated with Studies 1 and 2 were pre-processed and analyzed using corresponding procedures.

MIDI keystroke data were pre-processed in MATLAB R2017a using custom scripts, and all statistical tests were performed in RStudio v1.2.1335 running R v3.6.0. Specifically, all data were submitted to ANOVAs implemented using the *R aov_ez* function in the *afex* package v.23–0, which uses Type-III Sum-of-Squares, and applies Greenhouse-Geiser correction for violations of sphericity for within-subject factors. Follow-up comparisons were implemented using the *emmeans* package v.1.3.5, which by default uses effects coding (contr.sum) with *afex* objects. The *emmeans* model was changed from the default univariate model to a multivariate model (afex_options(emmeans_model = “multivariate”)), which provides better correction for violations of sphericity (https://cran.r-project.org/web/packages/afex/afex.pdf). Plots were created using *afex_plots()* in the *afex* package and custom *R* code.

### Pre-processing

- *Onset extraction and ITI calculation.* Tap onsets were extracted from MIDI files (timestamps, pitch, velocity) and inter-tap intervals (ITIs) between successive taps were computed for each Test trial.
- *Trial exclusion.* Test trials in the Joint Learning condition were flagged for exclusion during online data acquisition if partners did not observe the correct turn-taking structure for the respective study. Trials were reassessed offline for turn-taking accuracy, and all trials flagged as inaccurate were subsequently excluded from analyses. Rare Joint and Solo trials in which technical error occurred during data acquisition or subjects did not follow instructions were flagged online by the experimenter and were also excluded from offline analyses. The mean number of excluded Joint trials per pair was 1.21 for Study 1 (range = 0–3, median = 1), and 1.67 (range = 0–3, median = 2) for Study 2. The mean number of excluded Solo trials per pair was 0.63 (range = 0–2, median = 0.5) for Study 1 and 0.83 (range = 0–2, median = 1) for Study 2. It should be noted that pairs therefore repeated more trials in the Joint than Individual tasks. However, because this difference introduced a bias towards the null hypothesis (that Joint trials should not be more challenging for coordination than Individual trials), differences in trial number were not accounted for in subsequent analyses.
- *ITI outlier exclusion.* Rare ITI outliers (Study 1: Joint mean = 2.44%, range = 0.56–6.5%, Individual mean, computed within-pair = 1.3%, range = 0.28–1.16%; Study 2: Joint mean = 2.84, range = 0.37–7.96%; Individual mean, computed within-pair = 1.33%, range = 0.56–4.35%) were identified and excluded from all analyses, excepting lag auto-correlations and analyses based on IOI contrasts, both of which require continuous time-series, and are susceptible to biases introduced by outlier replacement. ITI outliers were defined within-subject for the Individual task, and within-pair for the Joint task. ITIs for each subject/pair were aggregated across all Learning Blocks to form a single task-specific distribution, and outliers in each distribution were identified as values exceeding 3 scaled median absolute deviations from the median of a distribution (using *isoutlier.m* in MATLAB)^29^ . This method is less susceptible than traditional- mean-based methods to false negatives in outlier detection^30^. Excluded ITIs were replaced with *NaNs* and (*nanmean.m* in MATLAB was subsequently used for all averaging).

### Calculation and analysis of dependent measures

Three primary dependent measures were computed from ITI data: accuracy, precision, and lag autocorrelations. ITI accuracy and precision provide insight into participants’ overall ability to maintain the cued rate of ITI production, whereas lag autocorrelations capture finer-grained temporal patterns, such as serial dependence and rhythmicity between ITIs. Described below is the specific rationale for each dependent measure, as well as the method for calculation and analysis.

#### ITI accuracy

- *Rationale* ITI accuracy captures how well participants were able to maintain the cued target ITI (500 ms). Accuracy was measured as percent deviation from the target ITI. If participants exhibit learning, then accuracy should increase across Learning Blocks (reflected as decreased percent deviation from the target ITI) in both Individual and Joint Learning. Moreover, equivalent levels of accuracy should be observed between Individual and Joint tasks by the final experiment block, if partners learn to “perform as one”.
- *Calculation* To compute ITI accuracy, ITIs on each trial were expressed as absolute percent deviation from the target ITI of 500 ms i.e. (abs(500 − ITI)/500) * 100. Absolute percent deviation values were averaged across ITIs within each trial, and then across trials for each participant within each Learning Block/Task.
- *Analysis* Mean accuracy values for Study 1 were subsequently submitted to a 2 × 4 × 2 mixed ANOVA with within-subject factors of Task (Individual/Joint) and Learning Block (1–4), and the between-subjects factor of Pitch Predictability (Predictable/Random). Pair was the grouping (ID) variable. Mean accuracy values for Study 2 were submitted to a 4 × 2 repeated-measures ANOVA with factors of Task (Individual/Joint) and Learning Block (1–4), also with Pair as the ID variable.

#### ITI precision

- *Rationale* ITI precision captures the extent to which participants produced stable temporal intervals. ITI precision was assessed using the Coefficient of ITI Variation (*CV(ITI)*, see calculation below), which measures ITI variability as a proportion of ITI duration, therefore controlling for the relationship between speed and variability. ITI precision should increase across Learning blocks (reflected as decreased Coefficient of Variation) in both Individual and Joint tasks if participants exhibit learning. Moreover, equivalent levels of *CV(ITI)* should be observed between Individual and Joint tasks by the final experiment block, if partners learn to “perform as one”.
- *Calculation* *CV(ITI)* was computed on each trial as *SD*($IT{I}_{trial}$) / *Mean*($IT{I}_{trial}$), and then averaged across trials for each subject within task and Block.
- *Analysis* Mean *CV(ITI)* values for Study 1 were subsequently submitted to a 2 × 4 × 2 mixed ANOVA with the between-subjects factor of Pitch Predictability (Predictable/Random), and the within-subject factors of Task (Individual/Joint) and Learning Block (1–4). Pair was the ID variable. Mean *CV(ITI)* values for Study 2 were submitted to a 4 × 2 repeated-measures ANOVA with factors of Task (Individual/Joint) and Learning Block (1–4), with Pair as the ID variable.

#### Lag autocorrelations

**Rationale.** Non-experts in motor behaviours such as dance and music performance often display greater rigidity of domain-specific movements than experts^9,21,31^. Rigidity is often characterized by rhythmical patterning of temporal intervals between successive movements^5^. Specifically, previous work on finger-tapping indicates that rhythmicity is characterized by regular durational alternation between successive ITIs, such that participants alternately shorten and lengthen successive ITIs, resulting in a *long-short-long-short* or *short-long-short-long* pattern^5^.

If participants in the current study exhibit learning, then it would be expected that ITI rhythmicity should decrease over the course across Learning Blocks. Moreover, similar levels of rhythmicity should be observed between Individual and Joint tasks by the final experiment block if partners learn to “perform as one”. Finally, rhythmicity may arise as a function of turn-taking structure.

To capture temporal patterns arising from the distinct turn-taking structures in Studies 1 and 2, lag autocorrelations were computed at lags 1, 2, and 4. Lag 1 captures patterns between serial ITIs (similar to ITI contrasts), whereas lags 2 and 4 capture patterns spanning intervals of 2 and 4 ITIs, which respectively correspond to the turn-taking rate (lag 2) and turn cycle duration (lag 4; time to complete a full turn cycle where each partner completes their turn once) in Study 2. Therefore, autocorrelations at lags 2 and 4 should differ between studies if turn-taking structure gives rise to distinct temporal patterns. Autocorrelations at lag 1 should simply confirm patterns revealed by ITI contrasts. Lag autocorrelation magnitude would be expected to decrease over the course of experiment blocks if learning is reflected in decreased long-range temporal patterns. Moreover, lag autocorrelations should not differ between Individual and Joint tasks by the final Learning Block if partners learn to “perform as one”.

- *Calculation* To compute lag autocorrelations, linear trends in ITI series were first removed from each trial prior (MATLAB function *detrend.m*), as linear drift can bias autocorrelation outcomes. Detrended ITI series were assessed for autocorrelations (MATLAB function *autocorr.m* in the Econometrics Toolbox), and the resulting autocorrelations were subsequently averaged across trials at lags 1, 2, and 4 for each subject within task and Block. Outlier ITIs were retained in autocorrelation analyses to avoid breaking serial dependencies between successive ITIs, which are critical to the lag structure.
- *Analysis* *Within-study comparisons.* Lag autocorrelations for Study 1 were subsequently submitted to a 2 × 2 × 4 × 3 mixed ANOVA, with between-subjects factor of Pitch Predictability (Predictable/Random), and within-subjects factors of Task (Individual/Joint), Learning Block (1–4), and Lag (1/2/4). Lag autocorrelations for Study 2 were submitted to a 2 × 4 × 3 repeated-measures ANOVA, with factors of Task (Individual/Joint), Learning Block (1–4), and Lag (1,2,4). Pair was defined as the ID variable for both ANOVAs.

*Between-study comparisons.* Lag correlations were also directly compared across studies. Trial-mean lag correlations from Studies 1 and 2 were submitted to a 2 × 2 × 4 × 3 mixed ANOVA, with between-subjects factor of Study (1/2), and within-subject factors of Task (Individual/Joint), Learning Block (1–4), and Lag (1/2/4). Pair was ID variable. Only Study 1 participants who completed the Random Pitch condition were included in this ANOVA so as to ensure equal samples across studies (*N* = 12 pairs per study), and identical task features excepting turn-taking structure.

## Results

### Learning effects revealed by ITI accuracy and precision across studies

#### ITI accuracy

##### Study 1

**Figure 2:**
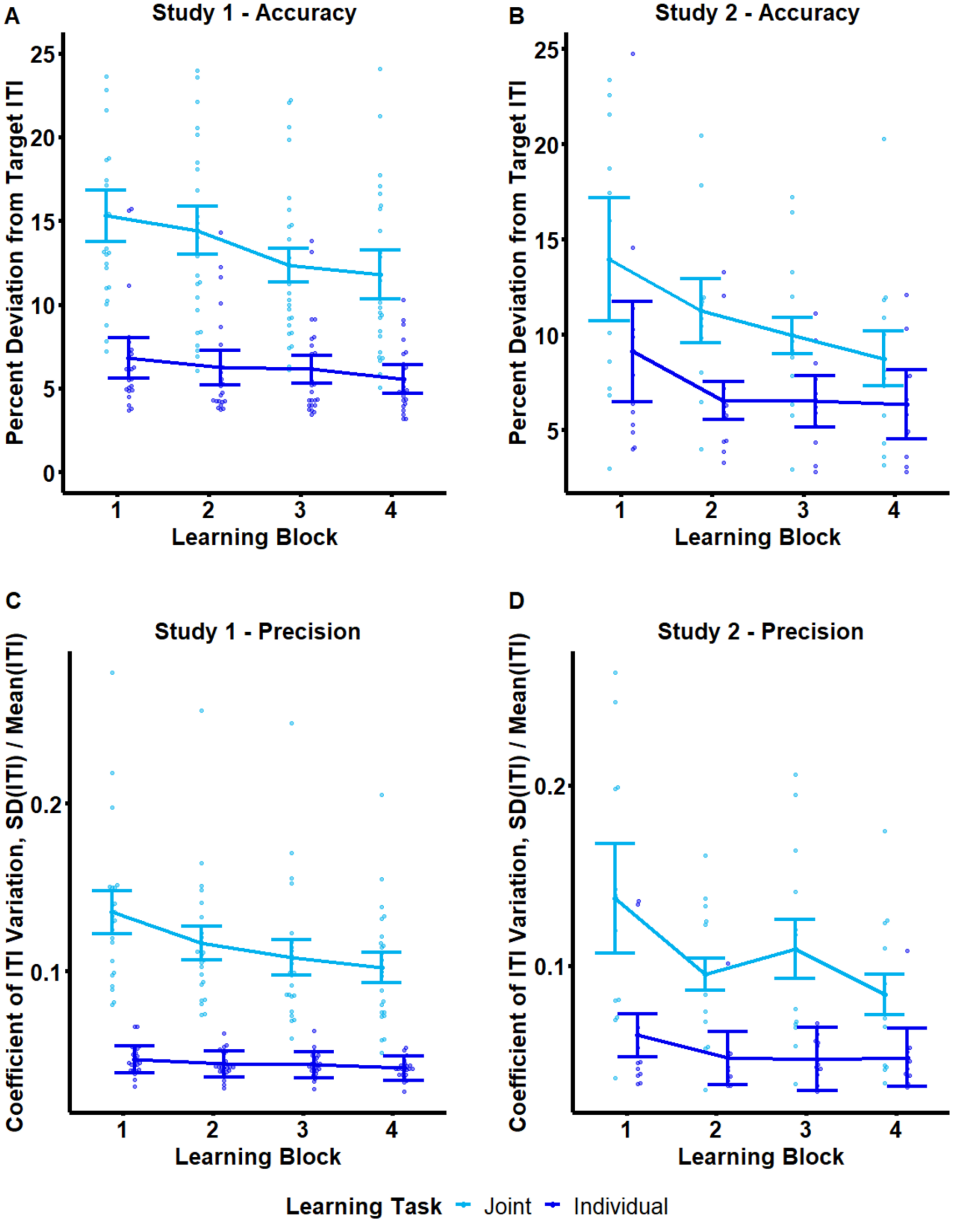
ITI Precision & Accuracy. Top row: Mean ITI accuracy (percent deviation from metronome-cued ITI) across Learning Blocks for Studies 1 (Panel A) and 2 (Panel B). Bottom row: Mean ITI precision (Coefficient of Variation, CV(ITI)) across Learning Blocks for Studies 1 (Panel C) and 2 (Panel D). In all panels, Individual and Joint tasks are displayed in dark and light blue respectively, and error bars reflect within-subject 95% Confidence Intervals (CIs).

Figure 2a shows changes in ITI accuracy across Learning Blocks for each task in Study 1. The 2 × 4 × 2 ANOVA on ITI accuracy revealed significant main effects of Learning Block, *F*(1.7782, 39.1195) = 9.0615, *p* = 0.0009, *ges* = 0.0468, Task, *F*(1.0000, 22.0000) = 90.6916, *p* =  < 0.001, *ges* = 0.4307, and a significant Task x Learning Block interaction, 2.2793, 50.1436) = 4.2849, *p* = 0.0154, *ges* = 0.0159. No other effects or interactions were significant (all *p*’s > 0.05).

Follow-up linear contrast analyses revealed significant linear effects of Block on ITI accuracy in both the Individual Task, *t*(22.0000) = − 2.4390, *p* = 0.0233, and the Joint Task, *t*(22.0000) = − 3.6430, *p* = 0.0014. Negative contrast estimates were observed in both Tasks (Individual: − 3.8520; Joint: − 12.5927), indicating improved ITI accuracy across Learning Blocks (reduced percent deviation from the target ITI). A linear interaction contrast (contrast of contrasts) revealed a stronger linear effect in the Joint than the Individual task, *t*(22.0000) = 2.7859, *p* = 0.0108, indicating more improvement across blocks in Joint relative to Individual Learning. However, partners in the Joint task did not reach Individual accuracy levels: Pairwise comparisons (Tukey-corrected) indicated that accuracy was significantly lower for the Joint relative to the Individual task across all Learning Blocks (all *p*’s < 0.001).

##### Study 2

Figure 2b shows changes in ITI accuracy across Learning Blocks for each task in Study 2. The 4 × 2 repeated-measures ANOVA on ITI accuracy revealed significant main effects of Learning Block, *F*(1.7314, 19.0459) = 10.5204, *p* = 0.0012, *ges* = 0.1068, and Task, *F*(1.0000, 11.0000) = 19.2066, *p* = 0.0011, *ges* = 0.1627. No other effects or interactions were significant (all *p*’s > 0.05).

Follow-up linear contrast analyses revealed a significant linear effect of Block on ITI accuracy in the Joint task, *t*(11.0000) = − 3.4566, *p* = 0.0054. Negative contrast estimates were observed in both Tasks (Individual: − 8.3332; Joint: − 16.9328), indicating improvement in accuracy across Learning Blocks in both Individual and Joint Learning. No linear interaction contrast was computed because, unlike Study 1, the ANOVA on ITI accuracy did not yield a significant interaction between task and Learning Block.

#### ITI Precision

##### Study 1

Figure 2c shows changes in ITI precision across Learning Blocks for each task in Study 1. The 2 × 4 × 2 ANOVA on ITI precision also revealed significant main effects of Learning Block, *F*(2.4035, 52.8760) = 14.9768, *p* =  < 0.001, *ges* = 0.0609, Task, *F*(1.0000, 22.0000) = 102.3690, *p* =  < 0.001, *ges* = 0.6163, and a significant Task × Learning Block interaction, 2.5849, 56.8689) = 10.0374, *p* =  < 0.001, *ges* = 0.0347. No other effects or interactions were significant (all *p*’s > 0.05).

Follow-up linear contrast analyses again revealed significant linear effects of Block on ITI precision in both the Individual task, *t*(22.0000) = − 4.1073, *p* = 0.0005, and the Joint task, *t*(22.0000) = − 5.1224, *p* =  < 0.001. Both Tasks showed negative contrast estimates (Individual: -0.0162; Joint: − 0.1072), reflecting decreased variability and improved ITI precision across Learning Blocks. An interaction contrast revealed a stronger linear effect in the Joint than the Individual task, *t*(22.0000) = 4.5422, *p* = 0.0002. However, partners in the Joint task did not reach Individual precision levels: Pairwise comparisons indicated that precision was significantly lower for the Joint relative to the Individual task across all Learning Blocks (all *p*’s < 0.001).

##### Study 2

Figure 2d shows changes in ITI precision across Learning Blocks for each Task in Study 2. The 4 × 2 ANOVA on ITI precision also revealed significant main effects of Learning Block, *F*(1.3024, 14.3259) = 12.5350, *p* = 0.0019, *ges* = 0.0856, Task, *F*(1.0000, 11.0000) = 25.4283, *p* = 0.0004, *ges* = 0.3124, and a significant Task × Learning Block interaction, *F*(2.0152, 22.1668) = 5.6276, *p* = 0.0104, *ges* = 0.0347.

Follow-up linear contrast analyses revealed significant linear effects of Block on ITI precision in Joint, *t*(11.0000) = − 3.8778, *p* = 0.0026, but not Individual Learning (*p* = 0.1139). Joint Learning showed negative contrast estimates, − 0.1452 (reflecting decrease in percent deviation from the target ITI), indicating improved ITI accuracy across Learning Blocks. An interaction contrast confirmed a stronger linear effect in the Joint than the Individual task, *t*(11.0000) = 2.6729, *p* = 0.0217. However, partners in the Joint task did not reach Individual precision levels: Pairwise comparisons indicated that precision was significantly lower for the Joint relative to the Individual task across all Learning Blocks (all *p*’s < 0.01).

It should be noted that, as can be observed in Fig. 2, variance of the Individual task accuracy data appears to be much smaller than the Joint data: The possibility that variability by definition increases when two people try to complete a task as one is an interesting question for future studies investigating how noise in motor timing changes from individual to joint motor tasks. To address the possibility that ANOVA outcomes were influenced by normality differences, ANOVAs were computed on log-transformed ITI accuracy and CV data. Significant effects and interactions were consistent with ANOVAs computed on un-transformed data. Therefore, the present manuscript reports parametric analyses of the un-transformed data.

#### Distinct rhythms of joint action across studies revealed by lag autocorrelations

##### Study 1

**Figure 3:**
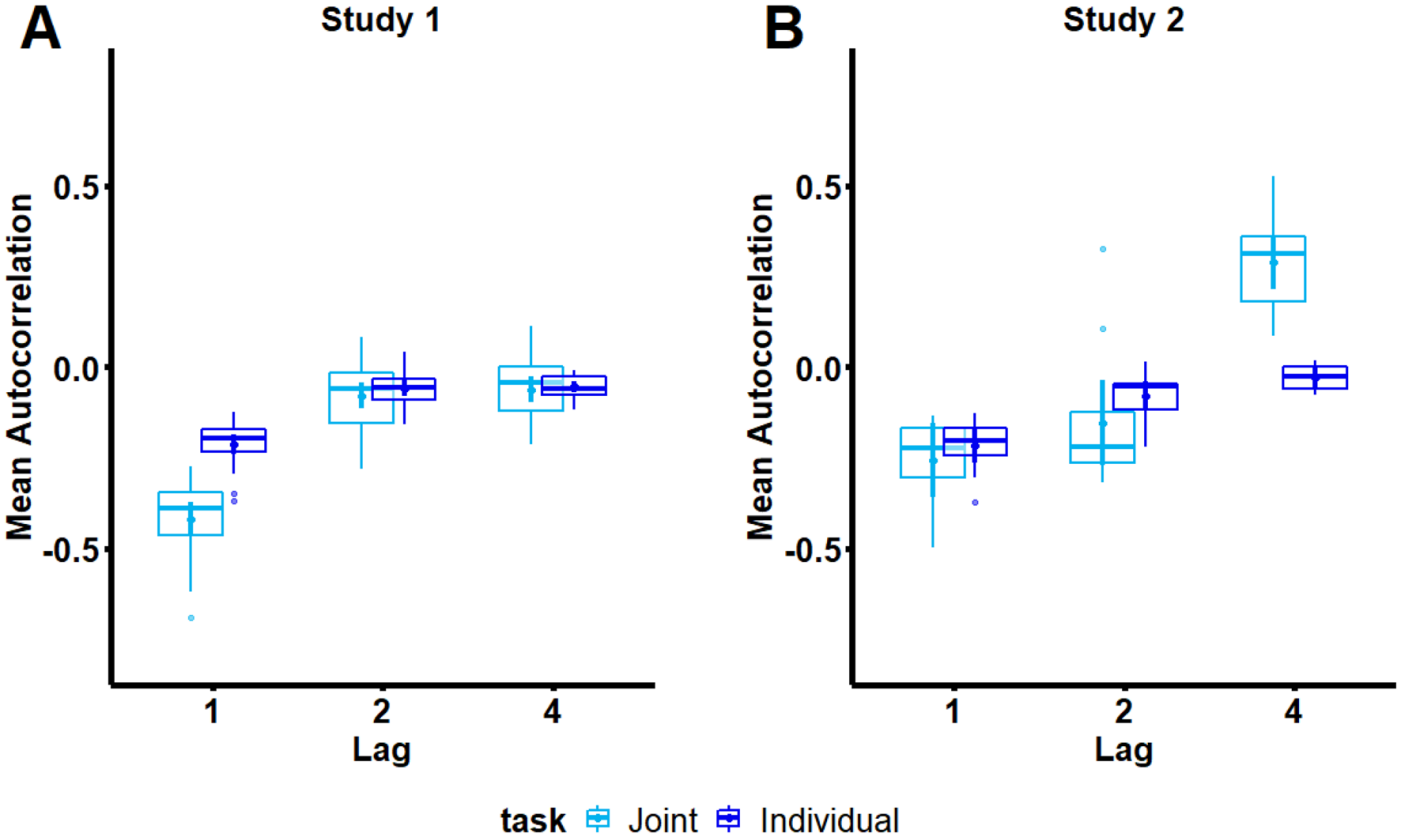
Autocorrelations at lags related to turn-taking. Boxplots displaying mean autocorrelations at lags 1, 2, and 4 for Study 1 (Panel A) and Study 2 (Panel B) for Individual (dark blue) and Joint (light blue) tasks. Boxplots display the median of each distribution (horizontal lines), where and lower and upper hinges reflect 25% and 75% around the median, and upper and lower whiskers reflect + /− 1.5 × the Inter-Quartile Interval for the distribution (default statistical settings in geom_boxplot() in the ggplot2 R package, as called from afex_plot).

Figure 3a displays mean ITI autocorrelations for Study 1. The 2 × 2 × 4 × 3 ANOVA on autocorrelations at lags 1,2, and 4 revealed significant main effects of Task, *F*(1.0000, 22.0000) = 71.9135, *p* =  < 0.001, *ges* = 0.1600, Lag, *F*(1.8301, 40.2630) = 122.4648, *p* =  < 0.001, *ges* = 0.6358, and a significant Task × Lag interaction, 1.6840, 37.0476) = 33.5599, *p* =  < 0.001, *ges* = 0.2036. No other effects or interactions were significant (all *p*’s > 0.05).

Significant interactions were followed up by pairwise comparisons using the *contrast()* call in the *emmeans* package (method = “pairwise”), with Tukey-correction applied in the case of multiple comparisons. Specifically, follow-up comparisons for the two-way interaction between Task x Lag revealed that the difference between Individual and Joint tasks was driven by significantly greater magnitude of negative autocorrelations at Lag 1 for the Joint task (*M* = − 0.4198, *SE* = 0.0225) relative to the Individual task (*M* = − 0.2128, *SE* = 0.0124) Learning, *t*(22.0000) =  − 9.1798, *p* =  < 0.001, Learning. In contrast, autocorrelations at lags 2 and 4 did not differ between Individual and Joint tasks.

##### Study 2

Figure 3b displays mean ITI autocorrelations for Study 2. The 2 × 4 × 3 ANOVA on autocorrelations at lags 1, 2, and 4 revealed significant main effects of task, *F*(1.0000, 11.0000) = 26.0066, *p* = 0.0003, *ges* = 0.0625, Lag, *F*(1.4069, 15.4754) = 49.0705, *p* =  < 0.001, *ges* = 0.5718, and a significant task x Lag interaction, *F*(1.6348, 2.8634, 17.9824) = 16.6268, *p* = 0.0002, *ges* = 0.3113. No other effects or interactions were significant (all *p*’s > 0.05).

Significant interactions were followed up by pairwise comparisons using the *contrast()* call in the *emmeans* package (method = “pairwise”), with Tukey-correction applied in the case of multiple comparisons. Specifically, follow-up comparisons for the two-way interaction between Task x Lag revealed that the difference between Individual and Joint Learning was driven by significantly more positive autocorrelations at Lag 4 for Joint (*M* = 0.2900, *SE* = 0.0386) relative to Individual Learning (*M* = − 0.0295, *SE* = 0.0103), *t*(11.0000) = 7.8939, *p* =  < 0.001. Autocorrelations at lags 1 and 2 were both negative (Lag 2 M = − 0.1547, *SE* = 0.0550), and did not differ between Individual and Joint Learning.

#### Cross-study comparison of temporal patterns arising from turn-taking structure

Autocorrelations at lags 1, 2, and 4 were compared across studies (Random Pitch pairs only) to quantify the extent to which distinct temporal patterns arise from turn-taking structure. Autocorrelations at lag-1 reflect the turn-taking structure of Study 1, whereas lags at 2 and 4 reflect the turn-taking structure of Study 2 (where lag-2 reflects the inter-turn interval, and lag-4 reflects a full turn cycle, i.e. the intervals across which both partners complete their turn).The 2 × 2 × 4 × 3 ANOVA on autocorrelations at lags 1, 2, and 4 revealed significant main effects of Study, *F*(1.0000, 22.0000) = 29.0498, *p* =  < 0.001, *ges* = 0.0756, and Lag, *F*(1.5572, 34.2588) =  < 0.001, *ges* = 0.5659, suggesting study-specific rhythmic patterns in ITI structure. Significant interactions were also observed between Study × Task, *F*(1.0000, 22.0000) = 54.4636, *p* =  < 0.001, *ges* = 0.0921, Study × Lag, *F*(1.5572, 34.2588) = 13.0538, *p* = 0.0002, *ges* = 0.1452, Task × Lag, *F*(1.6905, 37.1910) = 23.8794, *p* =  < 0.001, *ges* = 0.2240, and Study × Task × Lag, *F*(1.6905, 37.1910) = 10.7532, *p* = 0.0004, *ges* = 0.1150. Together, these significant interactions indicate that the rhythmic structure of ITI sequences captured by lags-1, 2, and 4 differ across Studies and Tasks.No other effects or interactions were significant (all *p*’s > 0.05).

It should be noted that there were two extreme lag2 autocorrelation values in the Joint task for Study 2; however, these data points were not removed, as they fell within the overall confidence intervals computed across lags.

To assess how the rhythmic structure of ITIs differed across studies and tasks, the significant three-way interaction between Study × Lag × Task was followed up by Tukey-adjusted pairwise comparisons between estimated marginal means using the *contrast()* call in the *emmeans* package (method = “pairwise”). These comparisons revealed that Lag 1 autocorrelations were significantly more negative in Study 1 (*M* = -0.4360, *SE* = 0.0343) relative to Study 2 (*M* = − 0.2573, *SE* = 0.0343) for Joint Learning only, *t*(22.0000) = − 3.6868, *p* = 0.0013. Additionally, Lag 4 autocorrelations were significantly more positive in Study 2 (*M* = − 0.0295, *SE* = 0.0090) relative to Study 1 (*M* = − 0.0440, *SE* = 0.0090) for Joint Learning only, *t*(22.0000) = − 7.3140, *p* =  < 0.001. No other pairwise comparisons between marginal means for levels of Study x Lag x Task were significant (all *p*’s > 0.05).

**Figure 4:**
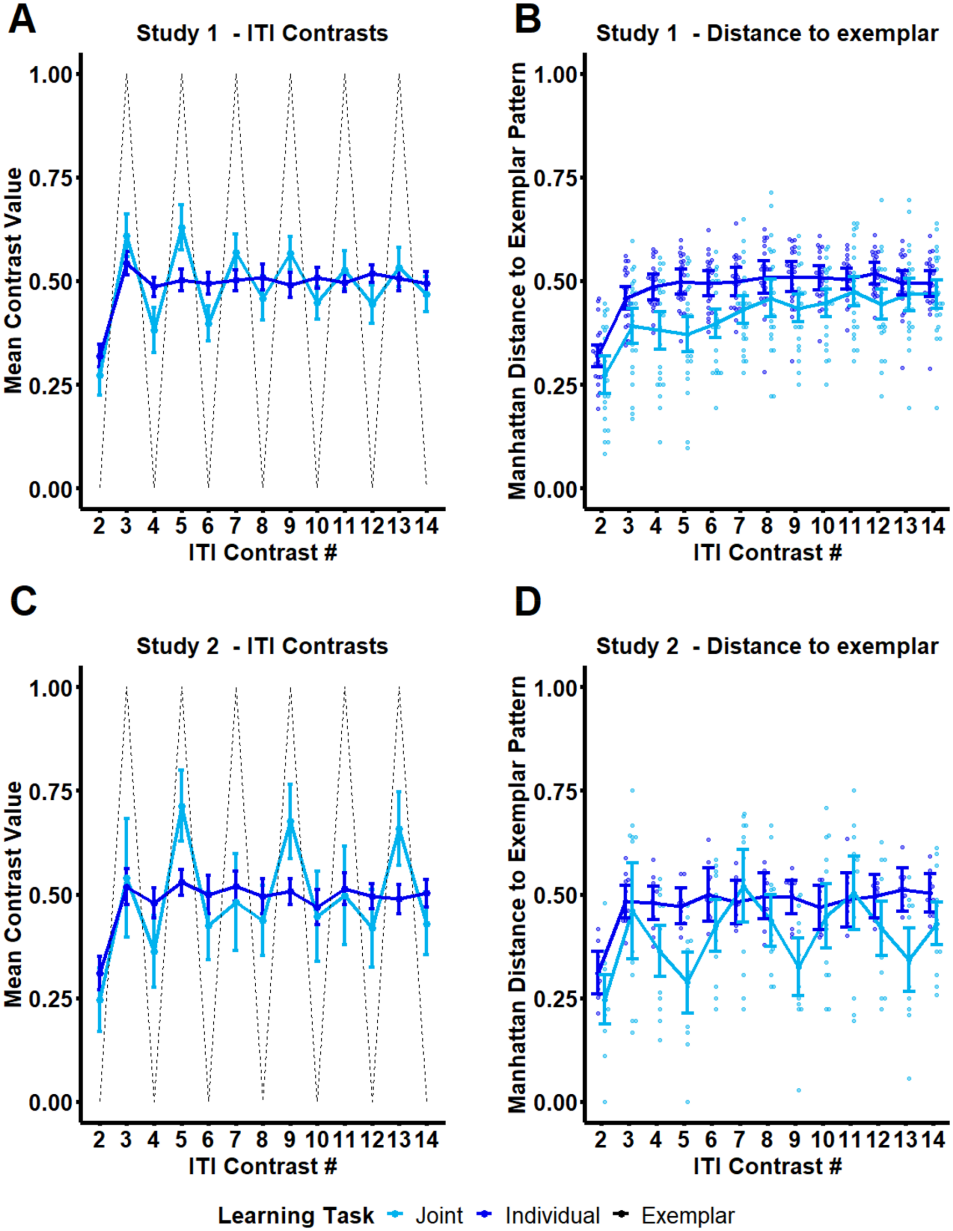
Illustration of study-specific rhythmic patterns. ITI contrast plots display patterns of durational change between successive ITIs, and clearly capture rhythmic patterns and changes in rhythmicity across ITIs in a sequence. The current figure displays mean ITI contrasts computed for Study 1 (Panel A) and Study 2 (Panel C) for Individual and Joint tasks, shown against an exemplar contrast pattern (black dotted line) representing perfect rhythmicity (negative lag-1 autocorrelation of 1.0). The mean geometric distance (Manhattan distance) to the exemplar at each ITI sequence position is also shown here for Study 1 (Panel B) and Study 2 (Panel C) to illustrate average changes in ITI rhythmicity over the course of a trial. Mean contrast values of .5 reflect no durational change between successive ITIs, mean contrast values above .5 reflect the same direction of durational change as the first pair of ITIs in the sequence, and values below .5 reflect the opposite direction of durational change as the first pair of ITIs. The figure clearly displays the enhanced rhythmicity of Joint relative to Individual ITI sequences in both studies, and that these rhythmicities depend upon turn-taking structure. Moreover, rhythmic patterns in Study 1 appear to change over time, reflecting within-trial learning. It should be noted that only ITI contrasts 2–14 are shown in each plot because the first ITI contrast on each trial was by default assigned a value of 1. Details of the methods for computing ITI contrasts are provided in the Supplementary Material.

The study-specific rhythmic patterns in the Joint task captured by lag autocorrelations can be clearly visualized using ITI contrasts, which code for directional change between successive ITIs. Figure 4 shows mean ITI contrasts across Studies and Tasks. Details on ITI contrast calculation are reported in the Supplementary Material.

## Discussion

The current study investigated how individuals learn to produce novel temporal sequences alone versus with a partner. A pseudo-musical sequence production task was used that allowed individuals with minimal musical experience to produce musical sequences without learning to play a musical instrument proficiently. In this task, participants tapped a single key on a piano keyboard at a cued rate, and each subsequent tap produced the next tone in a melody. Participants completed this task alone (Individual) and in turn with a partner (Joint), with the goal of producing stable temporal intervals at the cued rate. The joint turn-taking structure was manipulated across two studies such that turn-taking frequency was either high (Study 1, A-B-A-B turn-taking pattern) or low (Study 2, A-A-B-B pattern). In Study 1, the predictability of pitch sequences was also manipulated, such that pitch sequences were either predictable or random. Three main questions were addressed across studies: (1) Do the signatures of individual temporal sequence learning (increased temporal precision and accuracy, reduced serial dependency of timing errors) also characterize joint action learning? (2) Can partners achieve the same levels of temporal accuracy and stability as individuals over the same time-course of learning? (3) Does the predictability of sensory information associated with partners’ actions facilitate joint action learning? Findings are discussed below with regards to each of these questions.

### Turn-taking partners do not learn to perform “as one”

Participants displayed two classic signatures of learning in both studies and in both Individual and Joint tasks, namely improved temporal accuracy and precision with training. Improvements in accuracy were reflected by the linear reduction in percent deviation of observed inter-tap intervals (ITIs) from the cued interval (500 ms) across Learning Blocks. Improvements in temporal precision were reflected by the linear reduction in coefficient of ITI variation (*CV(ITI)*)—a measure of ITI variability that expresses variability as a proportion of duration—across Learning blocks.

Learning trajectories for both accuracy and precision were steeper for the Joint relative to the Individual task (indicated by contrasts of linear contrasts), giving rise to interaction effects between Block and Task (for precision in both studies, and for accuracy in Study 1). These differences in learning trajectories between tasks likely arise from the fact that accuracy and precision were closer to ceiling in the first Learning Block in the individual relative to the Joint task. Despite these improvements with training, partners in the Joint task were unable to achieve Individual levels of temporal precision and accuracy. Specifically, accuracy and precision were reduced across all Learning Blocks in the Joint relative to the Individual task, though in Study 1 Joint accuracy marginally approached Individual levels by the final block. It should be noted that, as can be observed in Fig. 2, variance of the Individual task accuracy data appears to be much smaller than the Joint data: The possibility that variability by definition increases when two people try to complete a task as one is an interesting question for future studies investigating how noise in motor timing changes from individual to joint motor tasks.

Taken together, learning to co-produce temporal intervals in turn with a partner—at high and low turn-taking frequencies—appears to be more challenging than producing intervals alone. Partners are worse than individuals at matching the tempo of a cued target interval and are less able to produce stable temporal intervals despite equal training, though they do show improvements over time. Reduced temporal precision and accuracy for co-produced relative to individually produced intervals may arise from differences in partners’ temporal error correction processes, as temporal error correction is known to vary widely between individuals^32^. Another possibility is that underlying oscillatory processes related to partners’ timekeeping—such as differences in partners’ natural frequencies of tapping—moderate the accuracy and stability with which partners co-produce temporal intervals. Future work should investigate these possible underlying mechanisms.

### Turn-taking structure is reflected in rhythmic patterns that emerge during joint learning

Lag autocorrelation findings from the current study reveal that joint action partners produce rhythmic patterns of error typically associated with novice motor behaviors. In Study 1, partners displayed an alternating long-short (or short-long) pattern of ITIs, captured by a negative lag1 autocorrelation and illustrated by ITI contrasts plots. This pattern was largely attenuated for individually produced sequences, and visualizations of ITI contrasts indicate that the pattern diminished across ITIs within trials, suggesting within-trial learning. In Study 2, partners also displayed rhythmic patterns of ITIs at the study-specific turn-cycle interval—namely at a lag of 4 ITIs. This pattern was not present for individually produced sequences and is also clearly illustrated by ITI contrast plots.

Direct statistical comparisons of lag autocorrelations between studies confirmed that rhythmic patterns arising in jointly produced sequences reflects turn-taking structure. Specifically, autocorrelations were directly compared between studies at lags 1 (corresponding to the turn-taking structure in Study 1), 2 (corresponding to the turn-taking interval in Study 2), and 4 (corresponding to the interval at which full turn cycles repeated in Study 2, i.e. A–A–B–B). Jointly produced sequences in Study 1 displayed greater rhythmicity at lag-1 (more negative lag-1 autocorrelations) than Study 2, with no between-study difference at lag-1 for individually produced sequences. In contrast, jointly produced sequences in Study 2 showed more pronounced rhythmicity at lag-4 (more positive lag-4 autocorrelations) than in Study 1, with no between-study difference in lag-4 autocorrelations for individually produced sequences. Together, both studies revealed specifically enhanced rhythmicity in jointly produced sequences at the interval of a full turn-cycle (A-B for Study 1, A-A-B-B for Study 2).

Interestingly, lag-2 autocorrelations (corresponding to the inter-turn interval for Study 2) did not statistically differ between studies in Joint or Individual tasks, suggesting that turn-taking specific temporal patterns arise at the level of turn cycles rather than turn intervals. It should be noted that two extreme lag-2 autocorrelation values in the Joint task for Study 2 may have reduced the possibility to detect study-specific rhythms at this lag; however, these data points were not removed, as they fell within the overall confidence intervals computed across lags.

In sum, turn-taking with a partner gives rise to rhythmic patterns that span across turn-taking cycles, i.e. the interval over which both partners complete one full turn. These temporal patterns are suggestive of motor grouping at the level of turn cycles. Motor grouping has been shown to facilitate acquisition of complex motor sequences^33^. Therefore, partners may have attempted to optimize performance in the Joint task by superimposing grouping patterns at the level of turn cycles, either intentionally or unintentionally. It should be noted that turn-taking specific rhythmicites showed difference signs between studies: Lag-1 autocorrelations were negative in Study 1, whereas lag-4 autocorrelations were positive in Study 2. These differences in sign may be related to the influence of local error correction mechanisms on lag-1 autocorrelations but not lag-4 autocorrelations.

It should also be noted that autocorrelations did not change systematically across blocks in a way that indicated learning (i.e. reduced rhythmicity over time), so it is unclear whether these grouping patterns might become fully attenuated with sufficient training, as partners become more proficient at co-producing temporal intervals. Future work should aim to increase trial length and overall number of trials to assess whether the observed rhythmicities arising from turn-taking structure attenuate with time, as partners become more proficient at coordination. Moreover, future work should assess whether enhanced rhythmicity at early stages of joint action learning predicts more proficient coordination at later stages of joint learning, as would be predicted from studies of motor learning within individuals.

### Predictability of sensory outcomes does not facilitate temporal learning

Finally, the sensory outcomes of individuals’ actions did not appear to influence learning in either Individual or Joint tasks, as evidenced by Study 1. Specifically, participants showed the same levels of performance and similar patterns of rhythmicity for both Individual and Joint tasks, regardless of whether finger taps produced the same pitch sequence on each trial or different pitch sequences on each trial. This finding is counter to the hypothesis that predictable feedback associated with one’s actions should facilitate coordination. This initial hypothesis was based on prior work indicating that sensory feedback about a partner’s actions facilitates prediction^15^, and that novices are better at learning how to coordinate the timing of actions when their actions produce highly predictable sensory feedback^20^.

The observed pattern of results may be explained by recent work suggesting that learning is unaffected by sensory feedback so long as it does not violate spatial contingencies between sound and movement (e.g. ascending motion with descending pitch)^20^. In the current task, there was no spatial contingency between sound and action (participants tapped a single key), and therefore the mere presence of sensory feedback may have served as a cue to a partner’s timing that facilitate joint action. This interpretation would also be consistent with prior work showing that unstructured feedback associated with joint actions such as single tones^12^ or haptic feedback^15^ are sufficient to facilitate learning to coordinate the timing of actions with a partner. Future work should aim to test this hypothesis by removing auditory feedback associated with joint actions in the present paradigm. If auditory feedback presence was essential for learning to coordinate actions, then coordination should break down in the absence of feedback, suggesting that. feedback presence but not content is critical for joint action learning.

It is also possible that the use of pentatonic pitch sequences—which do not give rise to strong harmonic expectancies due to the presence of purely consonant intervals—in the present studies may have prevented participants from developing strong pitch expectancies for Predictable melodies. Had Predictable melodies been composed using scales that generated stronger expectancies, then these expectancies may have given rise to stronger temporal predictions about upcoming sequence events in Predictable relative to Random sequences. Future work should aim to determine whether Predictable melodies that generate stronger pitch expectancies facilitate learning to predict a turn-taking partner’s actions relative to Random pitch sequences. Perhaps the influence of pitch expectancies on learning would be greatest in individuals with high scores on musicality indices, who may be more sensitive to pitch expectancy than individuals with lower musicality; future work should also aim to investigate how pitch sensitivity might moderate the influence of pitch predictability on learning to coordinate auditory-motor sequences with a partner.

## Conclusions

Taken together, the current work provides one of the first investigations into how partners learn to co-produce temporal sequences in turn-taking, utilizing a novel paradigm that allows for direct comparison between individual and joint sequence learning. Findings from both studies revealed that partners display classic signatures of individual sequence learning when learning to co-produce temporal intervals: increased temporal precision and accuracy with training. Partners did not, however, perform “as one”: they did not achieve individual levels of performance with regards to precision, accuracy, or serial dependence between successive intervals. And, most strikingly, partners superimposed unique rhythms onto joint sequences that were not present during individual learning, and which were related to the turn-taking structure.

Rhythmicity is a hallmark of novice motor behaviour, and is often interpreted as a signature of poorly calibrated error correction within-individuals^10^. The current studies are the first to demonstrate that rhythmicity also characterizes *jointly*-produced temporal sequences. Whether these rhythmicities are driven by an emergent joint timekeeping system between partners—and how this timekeeping system might be influenced by individual timekeeping processes—is an important question for future models of joint action coordination, and an obvious next step.

## Supplementary Information

Supplementary Information.

## Data Availability

The datasets generated during and/or analysed during the current study are available in the Zenodo repository, 10.5072/zenodo.1165461.
