# Supplementary material for: The spontaneous emergence of rhythmic coordination in turn taking
Source: Sci Rep. 2023 Feb 24;13:3259. doi: 10.1038/s41598-022-18480-6 (PMC9958099; doi:10.1038/s41598-022-18480-6)
Supplement: Supplementary file 1 — Supplementary Information. [file 41598_2022_18480_MOESM1_ESM.pdf]

Supplementary material - ITI contrast calculation

The dynamics of rhythmic patterns in ITIs were visualized (see Figure 4 in main text) by computing ITI contrasts, which code durational change between successive ITIs. To compute ITI contrasts, ITIs on each trial were first coded as a contrast vector of 1s and 0s: 1s represented all durational changes that went in the same direction as the first pair of ITIs in the trial (e.g. in the case of the *long-short-long-short* pattern, all ITI pairs that displayed lengthening), and 0s represented all durational changes that went in the opposite direction (e.g. in the case of the *long-short-long-short* pattern, ITI pairs that displayed shortening). Rare instances in which successive ITIs were of equivalent duration were assigned a contrast value of .5. This contrast coding method allowed for capturing rhythmic alternations of ITI duration independent of phase, i.e. regardless of whether trials started with a long-short-short-long pattern or short-long-short pattern. Outlier ITIs were retained for this analysis to preserve serial dependency between successive ITIs.

Contrast vectors were averaged across trials to obtain a stable representation of behaviour, and the extent of rhythmicity was quantified by comparison to an exemplar vector. The exemplar vector reflected a theoretical case of "perfect" rhythmicity, i.e. 1-0-1, corresponding to hypercorrection on every turn in Study 1 (Study 1 Exemplar Pattern). Similarity between observed and exemplar vectors was quantified as the L1 distance between vectors, a simple and commonly used method for assessing similarity between vectors containing categorical or nominal data,[31] and which has been widely used in the music informatics community to compute similarity between musical rhythms (for an overview see Toussaint et al.[32]). The L1 distance is a unitless measure, computed as the sum of the absolute differences between corresponding points in two vectors. A value of 0 indicates identical vectors (zero distance), and increasing values reflect increased distance (dissimilarity) between two vectors. Possible distances between any pair of points in observed and exemplar vectors therefore ranged from 0-1 using the current coding scheme; these elementwise distances measure how similarity to an exemplar changes over the course of a learning trial. Only ITI contrasts 2-14 are displayed in Figure 3, because the first ITI on each trial was by default assigned a value of 1.
